# Spontaneous rectosigmoid perforation at the watershed area of the Sudeck point in an apparently healthy toddler boy: a case report

**DOI:** 10.1186/s13256-023-04157-9

**Published:** 2023-10-09

**Authors:** Netsanet Solomon, Tilahun Habte, Seifu Alemu, Ayana Sori

**Affiliations:** Department of Surgery, Faculty of Public Health and Medical Sciences, Health Institute, Jimma University, P.O. Box 378, Jimma, Ethiopia

**Keywords:** Child, Rectal perforation, Idiopathic, Case report, Sudeck point, Colonic watershed areas, Peritonitis

## Abstract

**Background:**

Spontaneous colon perforation can be classified into stercoral and idiopathic. Stercoral type is associated with chronic constipation, thus it is rare in infants and children. The idiopathic type is sporadic and could occur at any age. Delay in diagnosing or treating idiopathic colon perforation is associated with high mortality and morbidity rates. There are few studies on rectal perforation related to other etiologies or past the neonatal period, and their effect on disease onset and prognosis are unknown.

**Case presentation:**

We report on a case of 2-year-and-5-month-old Oromo boy who presented with fever, diarrhea, vomiting, and progressive abdominal pain of 5-day duration. The boy underwent an exploratory laparotomy for suspected peritonitis and there was a single perforation of approximately 2.0 cm size in the anterior part of the upper one-third of rectum. The perforated rectum was repaired primarily and sigmoid divided diversion colostomy was carried out.

**Conclusion:**

It is important to be aware of idiopathic colon perforation in children, a rare but dangerous condition with high mortality and morbidity in cases of delayed diagnosis or management. Pediatricians and surgeons should consider colon perforation as a cause in children who present with abdominal distention and a history of diarrhea for more than 5 days.

## Introduction

Spontaneous bowel perforation not caused by disease or trauma, especially a colorectal perforation, is uncommon in children beyond the neonatal period and is difficult to diagnose preoperatively [1]. Spontaneous colon perforation (SCP) in pediatrics is usually encountered as necrotizing enterocolitis in the neonatal period, but is rare in infants and children without preceding conditions, including Hirschsprung’s disease, inflammatory bowel disease, connective tissue disorder, lymphoma, and infective colitis. It is more frequent in the elderly with history of constipation and older than 60 years of age [1–4]. There are studies that showed perforated Meckel diverticulum in a 3-day-old neonate and a 9-year-old female patient with perforated Crohn’s disease [5, 6].

There are very few studies on colorectal perforation related to other etiologies or past the neonatal period, and their effect on disease onset and prognosis are unknown. Here, we present a case of a 2-year-and-5-month-old male child who presented with rectal perforation without an identified etiology.

## Case presentation

A 2-year-and-5-month-old Oromo boy presented to the emergency department with complaints of abdominal cramp and distension for 5 days. He had a history of vomiting of ingested matters and blood mixed mucoid diarrhea of the same duration. There was a history of low grade fever for 4 days. The boy did not have urinary compliant and trauma to the abdomen. He did not take any drugs for the current compliant. He was from a rural area in Ethiopia, with frequent day to day contact with cattle. His parents were farmers by occupation and were illiterate. The family did not report neonatal history of prematurity, delayed passage of meconium, any chronic constipation, and diarrhea.

He weighed 11.8 kg with normal anthropometry ranges.

On examination, the child was irritable and vital signs were as follows: pulse rate (PR) of 130 beats per minute; blood pressure (BP) of 90/60 mmHg; respiratory rate (RR) of 26 breaths per minute; and temperature (T) of 38.8 °C. The abdomen was distended, moving with breathing, and tympanic to percussion. He had generalized abdominal tenderness more on the lower quadrants. Bowel sounds were active and there was no finding on digital rectal examination. The rest of physical examination was unremarkable.

### Investigations

Blood investigations were as follows: hemoglobin 14.5 g/dl, white blood cell (WBC) total counts 35,000/mm^3^; differential count: neutrophils 79%, lymphocytes 11%, eosinophils 10%. Renal and liver function and serum electrolyte tests were normal. Stool examination was non-revealing.

**Fig. 1:**
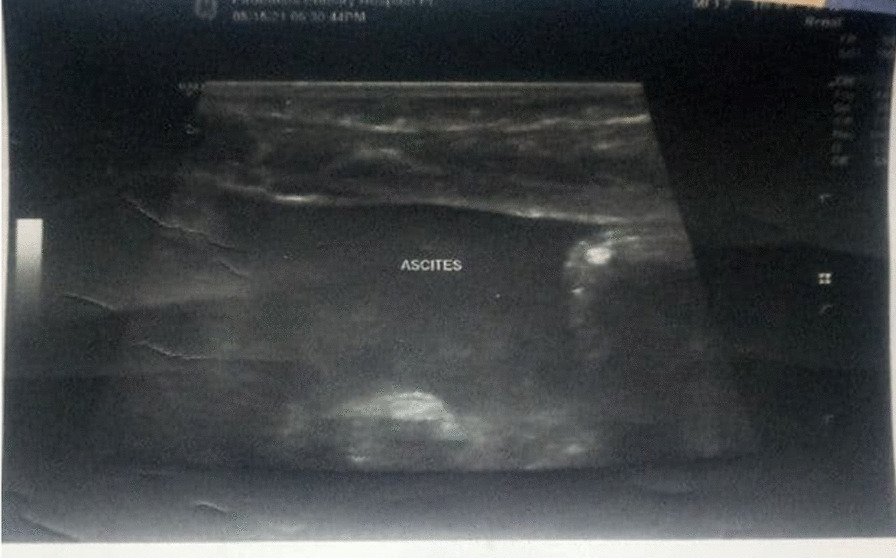
Abdominal ultrasound showing intraperitoneal collection with edematous bowel

Ultrasonography of the abdomen revealed small amount of peritoneal fluid collection in the paracolic gutter with floating debris and dilated edematous bowel (Fig. 1). X-ray was not taken for the patient.

With diagnosis of generalized peritonitis, the patient was resuscitated with intravenous fluids and nasogastric tube inserted for decompression, as well as urethral catheterization to monitor urine output.

### Therapeutic intervention

**Fig. 2:**
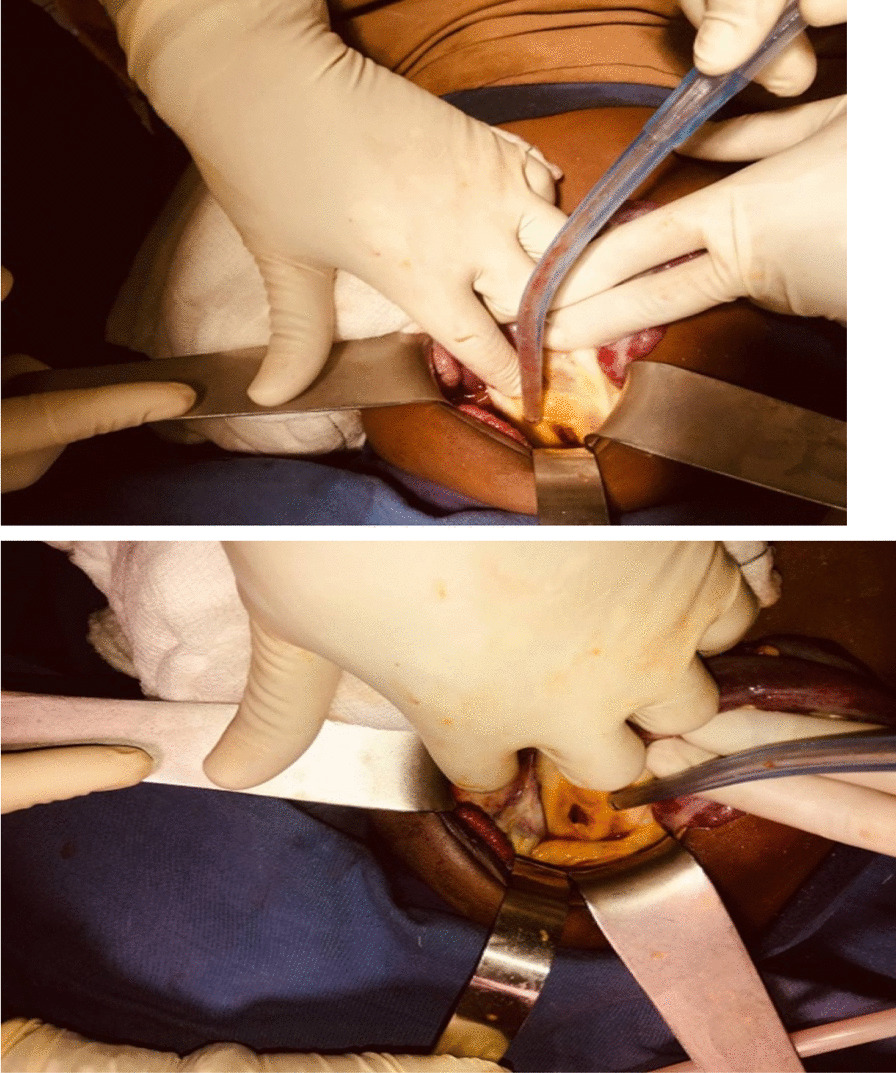
Intraoperative picture showing perforated rectum

After written informed consent was obtained from the child’s parents, the boy underwent emergency laparotomy for suspected peritonitis. On exploration, there was minimal fecal contamination more on pelvic peritoneum, which was irrigated and suctioned. Upon inspection, both small and large bowel were inflamed and edematous. There was a single perforation of approximately 2.0 cm size in the anterior part of upper one-third of rectum and the edge of peritoneum was necrotic (Fig. 2). After a thorough peritoneal lavage, a biopsy was taken from the edge of the perforation, and the necrotic edge debrided. The perforated rectum was repaired primarily and sigmoid divided diversion colostomy was carried out. Abdominal cavity washed with warm saline and closed in layer.

In the immediate postoperative period, the patient was treated with ceftriaxone (70 mg/kg/day intravenous every 12 hours) and metronidazole (30 mg/kg/day intravenous every 8 hours). Blood culture did not show the presence of any organism.

### Follow-up and outcomes

The postoperative period was uneventful. The patient was discharged on the 14th postoperative day with vital signs of PR 112 beats per minute, T 37.1 °C, and RR 26 breaths per minute. Pathology result showed scanty tissue with sheets of neutrophils and necrotic area, with conclusion of rectum with only necrotic tissue and sheets of neutrophils.

The patient was admitted for colostomy reversal after four visits in follow-up clinic and colostomy reversed and patient was discharged improved. He was appointed to pediatric surgery follow-up clinic, followed for more months, and was finally discharged from follow-up.

## Discussion

Colon perforation is a rare but life-threatening problem in the pediatric population, and has high mortality and morbidity rates if diagnosis or treatment are delayed [2, 7].

There are certain junctional weak points in the colonic marginal artery blood supply, known as watershed areas, which result from congenital incomplete development of anastomoses of the marginal arteries. These critical points in the marginal arcade are more vulnerable to consequences of ischemic injury than other parts of the marginal arterial arcade. Patient can develop perforations well localized in the rectosigmoid region at Sudeck point, in the splenic flexure region at Griffiths point, in the cecum-ascending colon junction, and a combination of these sites. The perforation happens on the antecolic area, as the blood supply is circumferential, this is the weakest point in the circumference of the colon tube. If the artery supplying the area of the atresia has been affected beyond the neighboring branch at these sites, there is little chance of an effective collateral circulation being established. There is an association of Hirschsprung disease or its variants in the colon distal to the site of perforation due to delay of craniocaudal migration of ganglion cells, and apparently these patients may not have overt symptoms and may look deceptively healthy. It is therefore suggested that all cases of pediatric colonic perforations should be biopsied from distal part of the colon as well as rectum to rule out Hirschsprung disease or its variants as an associated anomaly [8, 9].

SCP can be classified into stercoral and idiopathic types. Stercoral type is associated with chronic constipation, thus it is rare in infants and children. The idiopathic type is sporadic and could occur at any age. It is less common and shows better prognosis, possibly because of minimal fecal contamination [1]. Idiopathic colon perforation is more commonly found in the very old or the very young, especially premature infants, but is rare in children [7]. For our patient, we could not find previous recorded medical history, but his mother reported to have term pregnancy and the delivery was spontaneous vaginal delivery at a health institute.

Few studies have reported use of nonsteroidal antiinflammatory drugs (NSAIDs), even for a short period of time [2, 7], and non-typhoid *Salmonella* infection has been associated with spontaneous colon perforation [10–13]. Our patient did not use NSAIDs, but the specimen was not tested for bacteria. Another study found that spontaneous colon perforation in young children was a manifestation of Ehlers–Danlos syndrome [7, 14].

One study reported non-traumatic colon perforation commonly occurred in children with mean age of 2.22 ± 1.87 years, and 91.4% of them were under the age of 5 years [7]. Our patient’s case bore similarities to this finding, as the patient was a 2-year-and-5-month-old boy.

The most common symptoms and signs reported were fever (97.7%) and abdominal distention (93.1%), which our patient had before admission. In addition, the mean duration of symptoms prior to admission was 6.19 days [1, 7]. In our case, it was about 5 days.

Idiopathic perforation can occur at any part of the colon, but more commonly in the sigmoid colon, which is a vulnerable anatomical part in the vasculature and has been proposed to explain the colon perforation [7, 13]. In our patient’s case, the perforation occurred in the antimesenteric surface of rectum, which is rare in children. However, there is a report of isolated rectal perforation in a 3-year-old girl with enteric fever [12].

Colorectal perforation requires prompt surgical intervention because a good outcome can be expected when detected early with less fecal contamination. Surgical management for colon perforation depends on the time of onset, degree of peritonitis, and general condition of the patient [1, 2, 7]. We followed the same principle of management for our case by doing primary repair with diversion colostomy.

## Conclusion

It is important to be aware of idiopathic colon perforation in children, a rare but dangerous condition with high mortality and morbidity in cases of delayed diagnosis or management. Pediatricians and surgeons should consider colon perforation as a cause in children who present with abdominal distention and a history of diarrhea for more than 5 days.

## Data Availability

Data will be available upon request from the corresponding author.

## Abbreviations

BP: Blood pressure
Cm: Centimeter
Dl: Deciliter
HGB: Hemoglobin
kg: Kilogram
mg: Milligram
PR: Pulse rate
RR: Respiratory rate
SCP: Spontaneous colon perforation
T: Temperature
WBC: White blood cell
